# Functional Impairment of Mononuclear Phagocyte System by the Human Respiratory Syncytial Virus

**DOI:** 10.3389/fimmu.2017.01643

**Published:** 2017-11-27

**Authors:** Karen Bohmwald, Janyra A. Espinoza, Raúl A. Pulgar, Evelyn L. Jara, Alexis M. Kalergis

**Affiliations:** ^1^Millennium Institute on Immunology and Immunotherapy, Departamento de Genética Molecular y Microbiología, Facultad de Ciencias Biológicas, Pontificia Universidad Católica de Chile, Santiago, Chile; ^2^Departamento de Endocrinología, Facultad de Medicina, Escuela de Medicina, Pontificia Universidad Católica de Chile, Santiago, Chile

**Keywords:** human respiratory syncytial virus, dendritic cells, macrophages, infection, immunity

## Abstract

The mononuclear phagocyte system (MPS) comprises of monocytes, macrophages (MΦ), and dendritic cells (DCs). MPS is part of the first line of immune defense against a wide range of pathogens, including viruses, such as the human respiratory syncytial virus (hRSV). The hRSV is an enveloped virus that belongs to the *Pneumoviridae* family, *Orthopneumovirus* genus. This virus is the main etiological agent causing severe acute lower respiratory tract infection, especially in infants, children and the elderly. Human RSV can cause bronchiolitis and pneumonia and it has also been implicated in the development of recurrent wheezing and asthma. Monocytes, MΦ, and DCs significantly contribute to acute inflammation during hRSV-induced bronchiolitis and asthma exacerbation. Furthermore, these cells seem to be an important component for the association between hRSV and reactive airway disease. After hRSV infection, the first cells encountered by the virus are respiratory epithelial cells, alveolar macrophages (AMs), DCs, and monocytes in the airways. Because AMs constitute the predominant cell population at the alveolar space in healthy subjects, these cells work as major innate sentinels for the recognition of pathogens. Although adaptive immunity is crucial for viral clearance, AMs are required for the early immune response against hRSV, promoting viral clearance and controlling immunopathology. Furthermore, exposure to hRSV may affect the phagocytic and microbicidal capacity of monocytes and MΦs against other infectious agents. Finally, different studies have addressed the roles of different DC subsets during infection by hRSV. In this review article, we discuss the role of the lung MPS during hRSV infection and their involvement in the development of bronchiolitis.

## Introduction

### Mononuclear Phagocyte System (MPS): Background

Since the 1960s, the term MPS was defined as a family of cells differentiated from a common committed progenitor derived from the bone marrow (1–3). The MPS is composed of three major cell types, including monocytes, macrophages (MΦs), and dendritic cells (DCs) (2, 4). These cells share common morphologic and functional features, such as stellated form and the endocytic capacity (2). In addition, MPS cells express a heterogeneity of cell surface markers based on the tissue where they are located (3, 5, 6).

In the lungs, the cells of the MPS play a key role during host defense and homeostasis (7, 8). MΦs are found mostly in the alveolus adjacent to the epithelium and less frequently in the terminal airways and interstitial space, while most DCs are located in the pulmonary interstitium (7). Finally, this cellular system play critical roles in pulmonary host defense against viral pathogens, such as human respiratory syncytial virus (hRSV), which will be discussed in detail below.

#### Monocytes

Monocytes originate in the bone marrow from a common myeloid progenitor that is shared with granulocytes and macrophages (9). Furthermore, recruitment of monocytes is critical for an effective control and clearance of viral infections (10). It has been described that, in the bone marrow, the earliest monocytic precursor needs between two or three generations before becoming a mature monocyte that can be released into the peripheral blood. Once in the blood, these cells circulate for several days before entering the tissues and replenishing tissue macrophage populations (10). In the absence of an inflammatory process, it is thought that migration of monocytes into tissues is a random phenomenon (10). Once there, monocytes are able to differentiate into tissue-specific-resident phagocytes (8, 10). Monocytes can differentiate *in vivo* and *in vitro* into other myeloid cells, such as MΦs or DCs in response to cytokines including granulocyte-macrophage colony-stimulating factor and macrophage colony-stimulating factor (11–13).

In humans, monocytes can be classified into three groups according to molecular markers and their function: (1) classical (CD14^++^CD16^−^); (2) intermediate (CD14^++^CD16^+^); and (3) non-classical (CD14^+^CD16^+^) (12, 14). While classical monocytes exert a high myeloperoxidase and intermediate phagocytic activity, non-classical monocytes are important during inflammatory and antiviral responses (12, 15). On the other hand, murine monocytes are classified into two groups: (1) LyC6^low^ and (2) LyC6^High^ (12). While the LyC6^High^ subpopulation is responsible for the inflammatory and antimicrobial response (11, 12), LyC6^low^ monocytes contribute mainly to immune surveillance and to tissue repair (12).

#### Macrophages (MΦs)

Macrophages are characterized by their phagocytic capacity, which is required for the removal of cellular debris during tissue repair processes (16). MΦs are present in different tissues, such as the brain, bone marrow, lung, and liver, among others. During an inflammatory response, MΦs can migrate into various tissues from the peripheral blood (16).

Macrophages can be activated both by a microbial infection or by endogenous stimuli, which include inflammatory cytokines, such as IFN-γ, IL-4, and IL-13 (16). These cells can display two different activation profiles known as M1 (classic) and M2 (alternative) (17). The M1 MΦ subset displays higher antimicrobial, inflammatory and antigen-presenting capacity (17). Meanwhile, the M2 MΦ subset mainly displays anti-inflammatory activity (16–18). In addition, M1 MΦs are stimulated by IFN-γ, while the activation of M2 MΦs requires IL-4 and IL-13 (17, 19). Importantly, M1 and M2 polarization can be modulated by viral infections (20). The role of these cells during hRSV infection will be discussed below.

#### Dendritic Cells

Dendritic cells are specialized cells whose main function is to modulate the communication between the innate and acquired immune responses (21). These cells are considered as professional antigen-presenting cells (APCs) with a low phagocytic capacity, as compared to other cells of the MPS (7). In mice, two major subsets of DCs have been identified: (1) conventional DCs (cDCs) or “myeloid” DCs and (2) plasmacytoid DCs (pDCs) (Table 1). While cDCs locate mainly in lymphoid and non-lymphoid tissues, pDCs can be found in blood, lymph nodes (LN), and lymphoid tissues (22). Additionally, murine cDCs can be divided in two subtypes: CD103^+^ cDCs and CD11b^+^ cDCs (Table 1) (22, 23). Further, cDCs can be separated in lymphoid tissues in two subsets: CD8^+^ and CD11b^+^ cDCs. CD8^+^ cDCs express the CD8α transcript and protein, but not CD8αβ heterodimer, which is most commonly expressed by CD8^+^ T cells (Table 1) (22).

**Table 1:** Dendritic cell (DC) subsets, location, and their surface markers.

DC subset	Location	Surface markers	Reference
Mouse conventional DC (cDC) CD103^+^	Lymphoid organs and non-lymphoid organs	CD11c^+^	(22)
		MHC-II^+^	
		F4/80^−^	
		CD11b^−^	
		CD103^+^	

Mouse cDC CD11b^+^	Lymphoid organs	CD11c^+^	(22)
		MHC-II^+−^	
		CD103^−^	
		F4/80^−^ integrin	
		αEβ7^+^	
		CD11b^+^	

Mouse cDC CD8α^+^	Lymphoid organs	CD11c^+^	(22, 24)
		CD205^+^	
		MHC-II^+^	
		CD11b^−^	
		CD8α^+^	

Human myeloid DC CD1c^+^	Lymphoid organs	CD1c^+^	(14, 25, 26)
		CD11b^+^	
		CD11c^high^	
		BDCA-1^+^	
		CD206^+^	
		CD207^+^	

Human myeloid DC CD141^+^	Lymphoid organs	CD141^+^	(14, 25, 26)
		CD11c^+^	
		CD11b^−^	
		BDCA-3^+^	
		CLEC9A^+^	
		XCR1^+^	
		CD123^+^	

Plasmacytoid DC (pDC)	Blood, lymph nodes, and lymphoid tissues	CD11c^low^	(22)
		mPDCA-1^+^	

Human pDC	Blood and bone marrow	Lin^−^	(14, 25, 26)
		MHC-II^+^	
		CD123^+^	
		CD4^+^	
		CD303^+^	
		CD304^+^	

As for the case of human DCs, these cells can be divided into pDCs and myeloid DCs. These latter cells are additionally classified into two subsets: CD1c^+^ and CD141^+^ (Table 1) (14, 26). Here, CD1c^+^ and CD141^+^ are analogous to the mouse tissue-resident CD11b^+^ and CD103^+^ DCs, respectively (27).

With respect to the function of the various DCs subsets, cDCs display an increased ability of sensing tissue damage independent of their role in the capture, processing, and presentation of antigens (22). Equivalent to other MPS members, DCs are found in the lungs as is the case for CD103^+^ cDCs, CD11b^+^ cDCs, and pDCs subsets, which are distributed in the lamina propria (28). Furthermore, both subsets of cDCs (CD103^+^ and CD11b^+^) are found in the alveoli, allowing their migration to the mediastinal LNs (28). Consistent with this notion, it is known that lung DCs play an active role in the pulmonary pathogenesis caused by viral infection and asthma (28).

On the other hand, pDCs are able to secrete large amounts of type I IFN during viral infections and contribute to the maintenance of immune tolerance (22). The latter activity of pDCs is achieved through the expression of molecules, such as the inducible tolerogenic enzyme indoleamine 2,3-dioxygenase (IDO), the inducible costimulator ligand, and/or the programmed death 1 ligand. These molecules promote the expansion of regulatory T cell (Treg) and the suppression of self-specific and alloreactive lymphocytes (28–31).

## Epidemiology Features of hRSV Infection

Viral infections are the most important cause of acute lower respiratory tract infection (ALTRI), affecting mainly young children and the elderly (32, 33). Up to date, the main agent causing this pathology is the hRSV (33–37). Human RSV produces a broad spectrum of clinical manifestations, ranging from mild, such as rhinitis, to more serious symptoms that include bronchiolitis and pneumonia (38, 39). Clinical symptoms not only are due hRSV but also involve host risk factors, such as preterm birth, immunosuppression, congenital heart disease, and chronic lung disease (40–43). Importantly, it is known that almost 100% of children have been infected with hRSV before 2 years old, due to the fact that this virus is highly contagious and efficient at disseminating from one individual to the next (44, 45). Up to date, infections due to hRSV remain still as one of the most important global public health burdens affecting humans in all countries (45). Annually, approximately 33 millions new cases worldwide are associated to ALTRI caused by hRSV infection alone, affecting mainly children under 5 years old (33, 46). Moreover, hospitalization events due to a severe bronchiolitis or pneumonia caused by hRSV infection have increased and reached about a 10% of the total number of cases (46). Importantly, the annual cost of hospitalizations due to hRSV outbreaks is about 394 million USD, a situation that repeats every year (33, 47).

In young children, the immune system fails to establish a protective response against hRSV, which leads to frequent re-infections (33, 47–49). Lack of protective immunity is explained by an impaired induction of cellular and humoral immune memory after the primary exposure to hRSV (34, 39, 50). Furthermore, hRSV is capable of modulating phagocytic cell function, leading to the respiratory immunopathology that is a characteristic of the infection by this virus (44).

The most severe clinical manifestation caused by hRSV is bronchiolitis (51), which is mainly characterized by a distal bronchiole inflammation and obstruction, which reduces the airflow into small airways and impairs the exhalation capacity (52). All these alterations promote an abnormal lung function that is manifested as airway hyperexpansion, increased mucus production, atelectasis, and wheezing (52, 53). The bronchiolitis caused by hRSV infection also can produce long-term pathologies and sequelae, such as asthma and respiratory hyperreactivity (54).

After that hRSV encounters the airway epithelial cells (AECs), this virus gets in contact with innate immune cells, such as monocytes, MΦ, and DCs located at the lung tissue (10). These immune cells produce significant amounts of pro- inflammatory cytokines after a viral infection that is involved in controlling adaptive immunity by their interaction with helper T cells (10, 55). In addition to contributing to the clearance of microbial pathogens, monocytes and MΦs also play an important role as APCs to prime T lymphocytes (56). Consistently with this notion, monocytes, MΦs and DCs not only are involved during the acute inflammatory phase of hRSV-induced bronchiolitis but also contribute to the promotion of reactive airway disease caused by this virus (57–59).

## Monocytes are Required to Initiate the Immune Response Against hRSV

**Figure 1:**
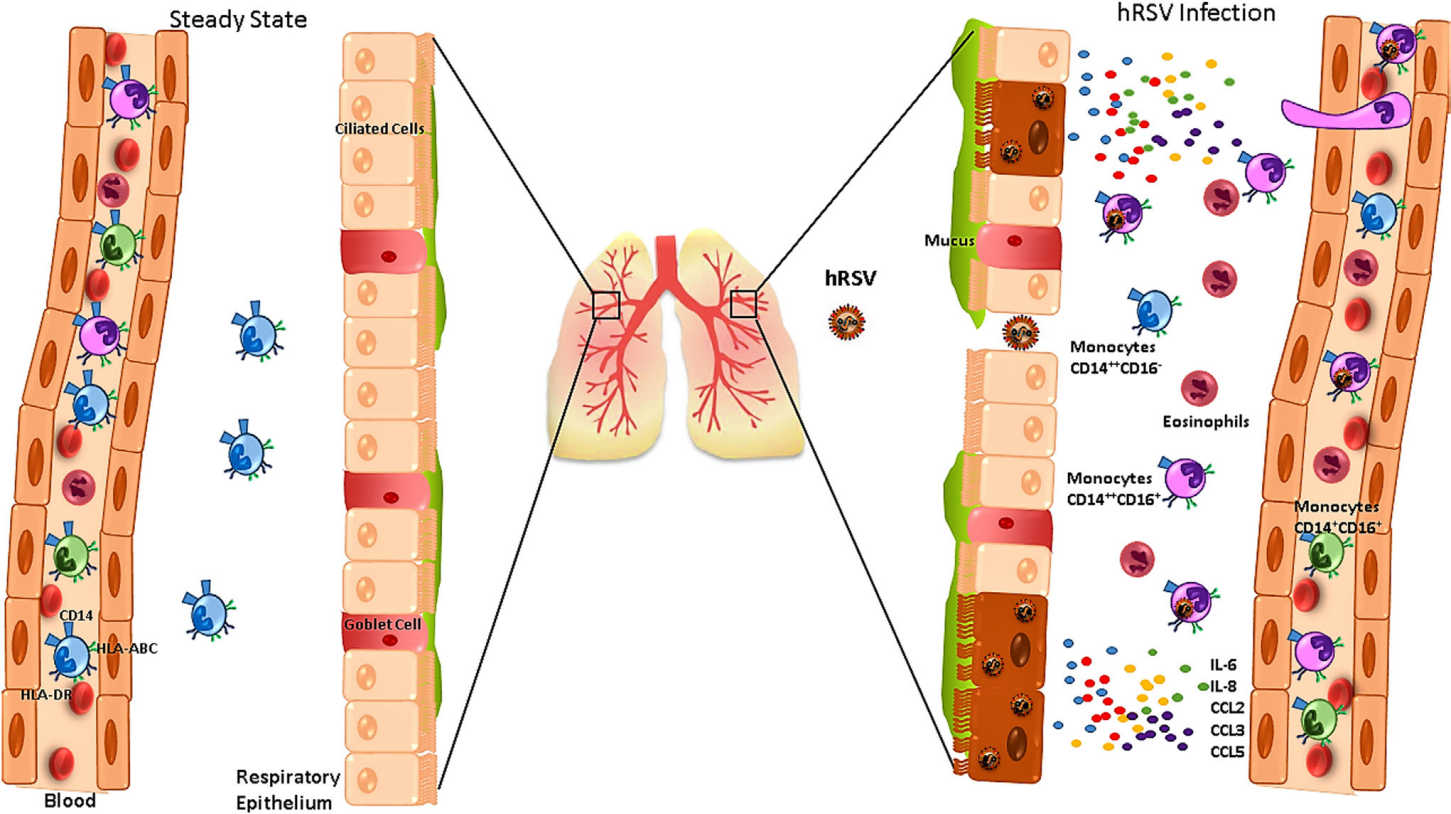
Human respiratory syncytial virus (hRSV) infection alters the function of monocytes and promotes their infiltration into the lungs. During the steady state (left), the main monocytes subset found in lungs are CD14^+^CD16^−^. During hRSV infection (right), infected airway epithelial cells secrete IL-6, IL-8, CCL2, CCL3, and CCL5, which promote monocytes and eosinophils infiltration into the airways. In addition, blood monocytes express increased levels of CD14 and HLA-ABC and reduced levels of HLA-DR, which is associated with severe bronchiolitis.

Monocytes are part of the first line of the host immune defense against viral pathogens (60). In response to infection with hRSV, human AEC-derived cell lines secrete cytokines and chemokines *in vitro*, including IL-6, IL-8, CCL2, CCL3, and CCL5 that promote the recruitment of monocytes and eosinophils to the site of infection (Figure 1) (60, 61). In addition, infection of BEAS2B cells (human lung epithelial cell line) with hRSV kept them from inhibiting the secretion of pro-inflammatory cytokines by monocytes, such as TNFα (62). During homeostasis, AECs are able to inhibit the function of inflammatory monocytes, a feature that is impaired in hRSV-infected AECs (62). Interestingly, monocytes can be directly infected by hRSV, reducing the expression of the intercellular adhesion molecule 1 and its ligand, the lymphocyte function-associated antigen 1, which alters the collaboration between monocytes and other immune cells (10, 63). These observations suggest that monocytes infected with hRSV can display a reduced capacity to induce a protective immune response against hRSV (10).

Patients infected with hRSV show a frequency increase for CD14^+^CD16^+^ monocytes in the blood (37). Furthermore, an increase of CD14 expression has been observed for all the monocyte subsets (Figure 1) (37, 64), suggesting that these cells can display an enhanced capacity to secrete cytokines and to migrate into the airways, probably to replace alveolar MΦ during hRSV infection (37, 64). Moreover, monocytes from hRSV-infected patients show a diminished expression of HLA-DR (Figure 1), correlating with disease severity (37, 65).

As mentioned above, hRSV infection causes bronchiolitis in children under 2 years old (66). Moreover, the pathology is worsened by some cytokines produced by monocytes during the early state of hRSV infection (67). Monocytes from of hRSV-infected patients presenting bronchiolitis in the convalescent stage of the infection secreted large amounts of IL-10 in response to stimulation with LPS and IFN-γ *in vitro* (67). Furthermore, authors showed a significant correlation between the monocyte-produced IL-10 and the number of wheezing episodes (10, 67).

Similar to MΦ and DCs, monocytes express TLR8 that promotes endosomal activation and IL-12p70 release upon binding to viral RNA (68). Monocytes derived from hRSV-infected infants displayed reduced expression of TLR8 during the acute phase of infection (68). Additionally, this study showed that monocytes from hRSV-infected infants produced reduced levels of TNF-α as compared to monocytes from healthy controls (68). Taken together, these results suggest that hRSV infection interferes with the normal expression of TLR8 and perhaps with the cytokines production that are important to initiate the immune response against hRSV (68).

The data relative to the role of monocytes during the immune response induced by the hRSV infection suggest that these cells are important to initiate the immunity against this pathogen. Further, monocytes are also involved in the development of bronchiolitis and the recurrent wheezing. Thus, it is likely that these cells could contribute to chronic respiratory sequelae caused by hRSV, such as asthma and airway hyperreactivity.

## Alveolar Macrophages (AMs) are Crucial to Control hRSV-Caused Disease

**Figure 2:**
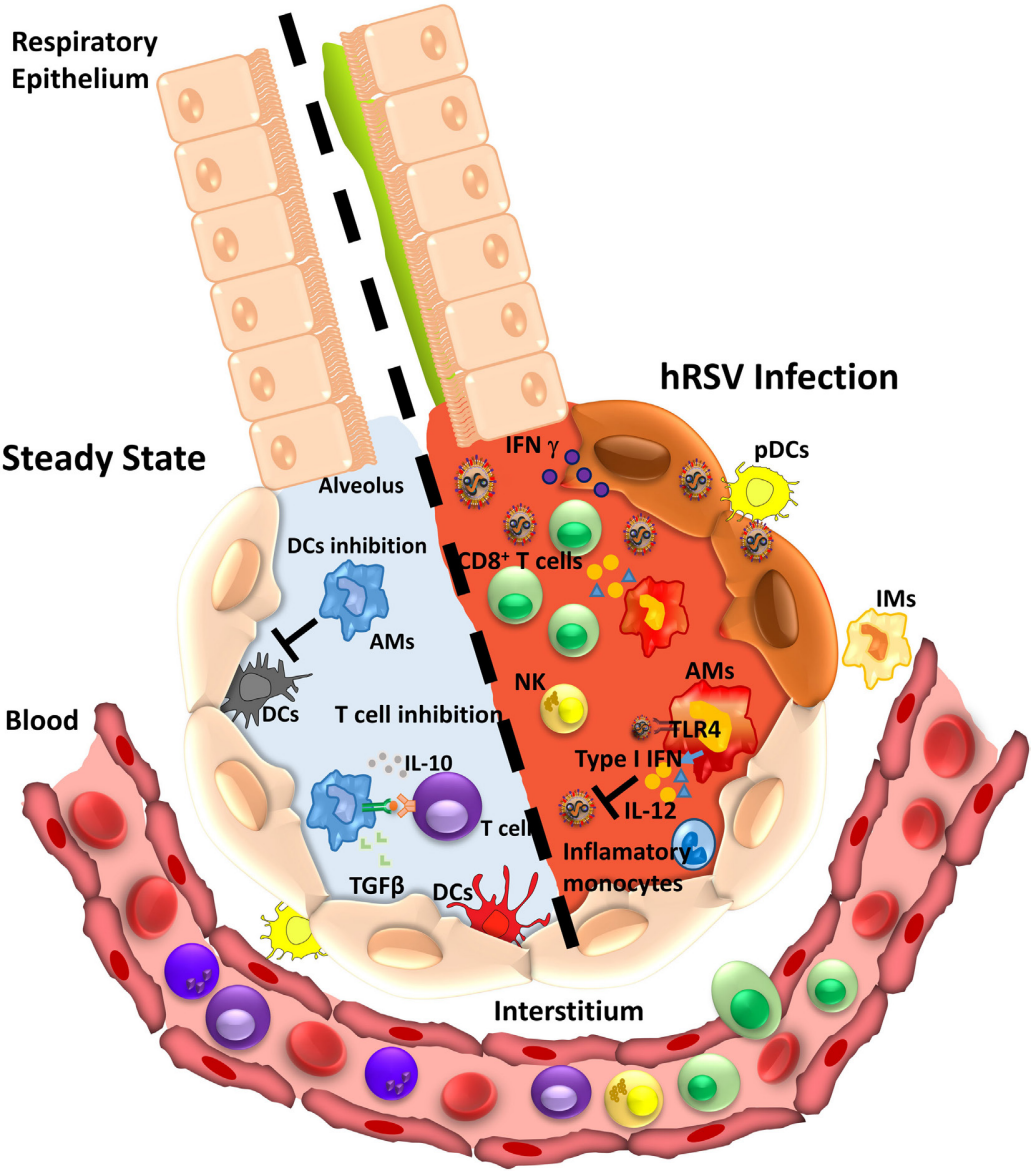
Alveolar macrophage (AM) function during homeostasis or infection by human respiratory syncytial virus (hRSV). During the steady state (left), AMs perform an anti-inflammatory function leading to immune tolerance. AM suppresses the response of T cell in an antigen-dependent manner and secretes cytokines as IL-10 and transforming growth factor-β (TGFβ). Also, AMs impair dendritic cells (DCs) activity inhibiting T cell presentation. Under hRSV infection (right), AMs can recognize the virus by TLR4 and respond by secreting type I IFNs that induce an antiviral state in epithelial cells. In addition, the secretion of pro-inflammatory cytokines promotes recruitment of inflammatory monocytes, CD8^+^ T cells, and NK cells that contribute with viral clearance.

Lung-resident macrophages consist of two distinct populations namely (1) AMs and (2) interstitial macrophages (IMs) (69). AMs locate in the luminal surface, while IMs reside in the interstitial space of the lung parenchyma (70). AMs are the most abundant phagocytic resident cells in the lungs, which uptake foreign particles, remove cellular debris, initiate immune responses against pathogens and contribute to restoring homeostasis in the lung epithelium (70). During the steady state (Figure 2), AMs can display an immunosuppressive effect by directly inhibiting the antigen-presenting function of lung DCs (71) or by inducing CD4^+^ T cell unresponsiveness in an antigen-specific manner (72). Furthermore, AMs can secrete several immunomodulatory molecules, such as IL-10, nitric oxide, prostaglandins, and transforming growth factor-β (Figure 2), which reduce inflammation in the lungs (73).

The role of AMs during hRSV infection has been characterized in murine models by depleting these cells through the administration of liposomes containing clodronate, a molecule that promotes the apoptosis of AMs (70, 74, 75). It was shown that AMs are crucial for the clearance of hRSV and for the control of lung inflammation (70). The depletion of AMs during hRSV infection leads to an increased viral replication and an exacerbated lung immunopathology (70). These results were consistent with a dramatic increase of neutrophils and inflammatory DCs recruitment to the lungs (70, 74). Then, in mice, AMs display a protective function during hRSV infection and contribute to attenuating lung inflammation and bronchiolitis triggered by this pathogen (76). Similar results were obtained in New Zealand Black (NZB) mice, which lack normal macrophage function and show an enhanced lung immunopathology upon hRSV exposure (76). Although the mechanisms responsible for the beneficial effect of AMs to control hRSV infection remain unknown, the available data suggest that the phagocytic and microbicidal capacity of AMs together with the secretion of type I IFN are the main elements contributing to the protection against this virus (76).

Alveolar macrophages are the main producers of type I IFNs in the airways during hRSV infection, even more than other cells, such as epithelial cells and pDCs (75). Type I IFN production by AMs is triggered by hRSV recognition and mediated by cytosolic mitochondrial antiviral signaling protein-coupled retinoic acid-inducible gene 1 (RIG-I)-like receptors (RLRs) (77). The type I IFN production promotes the monocytes-derived inflammatory cells recruitment (Figure 2), which further contributes to controlling hRSV infection and reducing lung pathology (77).

On the other hand, *ex vivo* experiments showed that hRSV infects both murine and human AMs (78). However, in both cases, the infection failed to lead to an increase of viral particle production (70, 79). These results suggest that the infection of AMs by hRSV might be abortive, allowing that AMs maintain a sentinel activity. Further, it is thought that abortive replication may allow AMs to resist the effects of hRSV NS protein, which inhibits the activity of RIG-I-like receptors (RLR) (77). Along these lines, AMs can restrict hRSV replication even in the absence of type I IFNs (75). However, the exposure of AMs to hRSV can result in a reduced phagocytic capacity during subsequent infections (79).

Moreover, AMs are essential for the activation of the early immune response against hRSV (70). Infection of human AMs by hRSV leads to the secretion of several pro-inflammatory cytokines, such as IL-6, TNF-α, IL-1β, and IL-8 (78, 80, 81). Conversely, similar experiments have described the secretion of IL-10 by these cells (82). The AMs response to hRSV is mediated mainly by the activation of NF-κB through recognition of non-replicative viral particles and surface viral proteins by TLR4 at early times postinfection (Figure 2) (83). Based on the available data about the role of TLR4 in the infection with hRSV, it has been hypothesized that in the beginning, surfactant protein A-opsonized hRSV can bind to TLR4 expressed on the surface of alveolar epithelial cells and AMs (84). Next, additional TLR4 and CX3CR1 molecules are recruited to the virus attachment site (84). Then, the hRSV F and G proteins interact with TLR4 and CX3CR1, respectively. Furthermore, both proteins interact with heparan sulfate structures (84), an interaction that is followed by the recruitment of caveolin-1 and the formation of caveolae with the subsequently recruitment of RhoA to the binding site (84). Moreover, it has been suggested that the binding of the hRSV F protein to TLR4 may activate the signaling pathway for this receptor and NF-κB translocation (84, 85). However, the role of TLR4 during the hRSV entry into target cells remains controversial, as well as the interaction with viral proteins that trigger the activation of the NF-κB pathway (86). A study performed in TLR4-positive cells (HEK 293 reporter cell lines) showed that infection with hRSV does not activate the NF-κB signaling pathway through the TLR4/MD-2/CD14 complex (87). However, in the context of the AM-directed immune response, studies have shown that in the absence of TLR4, the NF-κB signaling pathway is not activated (83) and that the latter is required for the polarization of MΦ toward the M2 phenotype (88). On the other hand, the establishment of this initial condition allows an effective lymphocyte recruitment and proper antiviral activity. Along these lines, human neonatal AMs infected with hRSV showed an impairment in the IFN-γ and IL-12 production (81). An inefficient secretion of IFN-γ has been associated with an increase of severe illness in infants (89). Considering that IFN-γ is necessary for the activation of AMs, a reduced production of IFN-γ in neonates has been observed to impair AMs activation, affecting the phagocytic capacity of these cells and exacerbating the hRSV-mediated bronchiolitis (90, 91). Also, an impaired AMs function reduces the T and NKT cells recruitment to the lungs, contributing to higher viral loads (91).

According with the data described above, AMs are important for the elicitation of an early immune response against hRSV, contributing to the viral clearance mainly mediated by the type I IFN secretion and the coordination of the adaptive response against this pathogen. Thus, an impaired function or absence of AMs can increase hRSV-induced bronchiolitis, both in mouse models and infants.

In contrast to the significant research efforts to understand the role of AMs during the infection with hRSV, the participation of IMs has only been poorly studied (92). Qi et al. evaluated the role of AMs and IMs in the production of IL-33 during hRSV infection (93). This study showed that the absolute number of IMs in lungs of hRSV-infected mice remained constant during the hRSV infection, in contrast to the increase observed for the absolute number of AMs (93). Furthermore, IMs from lungs of hRSV-infected mice showed an increase in the expression of both TLR3 and TLR7 mRNA (93). Considering that the number of IL-33-producing IMs in the lungs of mice was affected by hRSV infection, authors concluded that the IMs may not be the source of IL-33 during hRSV infection (93).

Therefore, additional studies are required to better understand the contribution of IMs to hRSV infection and pathogenesis.

## DCs as Commanders of the Immune Response During hRSV Infection

The infection with hRSV can induce different immune responses depending on the type of DC subset infected (94). According to this notion, it has been described that hRSV infection promotes CDs maturation by increasing the expression of CD80, CD86, CD40, and MHC-II in the lungs, which leads to a decrease of phagocyte function (95). Moreover, some studies in mice have reported that during the acute phase of hRSV infection, the frequency of mature DCs in the lungs is increased (95). On the other hand, it has been shown that hRSV has the capacity to infect and replicate inside DCs but in a non-productive manner (96–98). Importantly, it has been described that toll-like receptors expressed by DCs can interact with hRSV proteins. TLR-2 interacts with the viral fusion glycoprotein (F) and TLR-4 with both the F and the attachment G protein (99, 100). TLR4 activation promotes the secretion of IL-6 and TNF-α, as well as antigen cross-presentation *in vivo* and *in vitro* (101). Additionally, hRSV G glycoprotein interacts with DC- and L-SIGN, inducing both DC/L-SIGN-dependent and -independent phosphorylation of ERK1 and ERK2. As a result, DCs activation is impaired (102). This mechanism can be considered as a possible explanation for the reduced immunity induced by hRSV reinfections.

To understand as to how DCs become infected with hRSV, *in vitro* and *in vivo* experiments were performed in mouse models, which showed the contribution of Fcγ receptors (FcγRs), mainly FcγRIII, to infection by this virus (103). Human RSV-infected FcγRIII KO mice showed reduced airway inflammation as compared to infected wild-type mice, suggesting that FcγRIII plays a pro-inflammatory role during hRSV infection (103). On the other hand, it is known that hRSV infection induces only weak immune memory in the host (50). To understand this phenomenon, a possible impairment of the immunological synapsis between hRSV-infected DCs and T cells was evaluated *in vitro*. It was observed that hRSV infection of DCs not only impaired the assembly of the immunological synapsis with T cells but also the activation of naïve antigen-specific T cells (50, 104). The hRSV virulence factor that seems responsible for the inhibition of immunological synapse assembly is the nucleoprotein (N) (104). The N protein was found on the DCs membrane and by itself could interfere with the assembly of the immunological synapsis (104). Further, the N protein was located nearby to the TCR–pMHC complexes at the DC-T cell synapse interface (50, 104).

On the other hand, it has been reported that the mTOR protein on bone marrow-derived dendritic cells (BMDCs) plays an important role during hRSV infection (105). According to this notion, mTOR inhibition by rapamycin in hRSV-infected BMDCs decreased the number of CD8^+^CD44^high^ T cells, suggesting that mTOR is necessary for the proliferation of the T cell memory subset (105). Moreover, the treatment of the hRSV-infected BMDCs with rapamycin did not affect maturation and increased the survival when DCs were cocultured with T cells, suggesting that this phenomenon requires the contact of both cell types (105).

**Figure 3:**
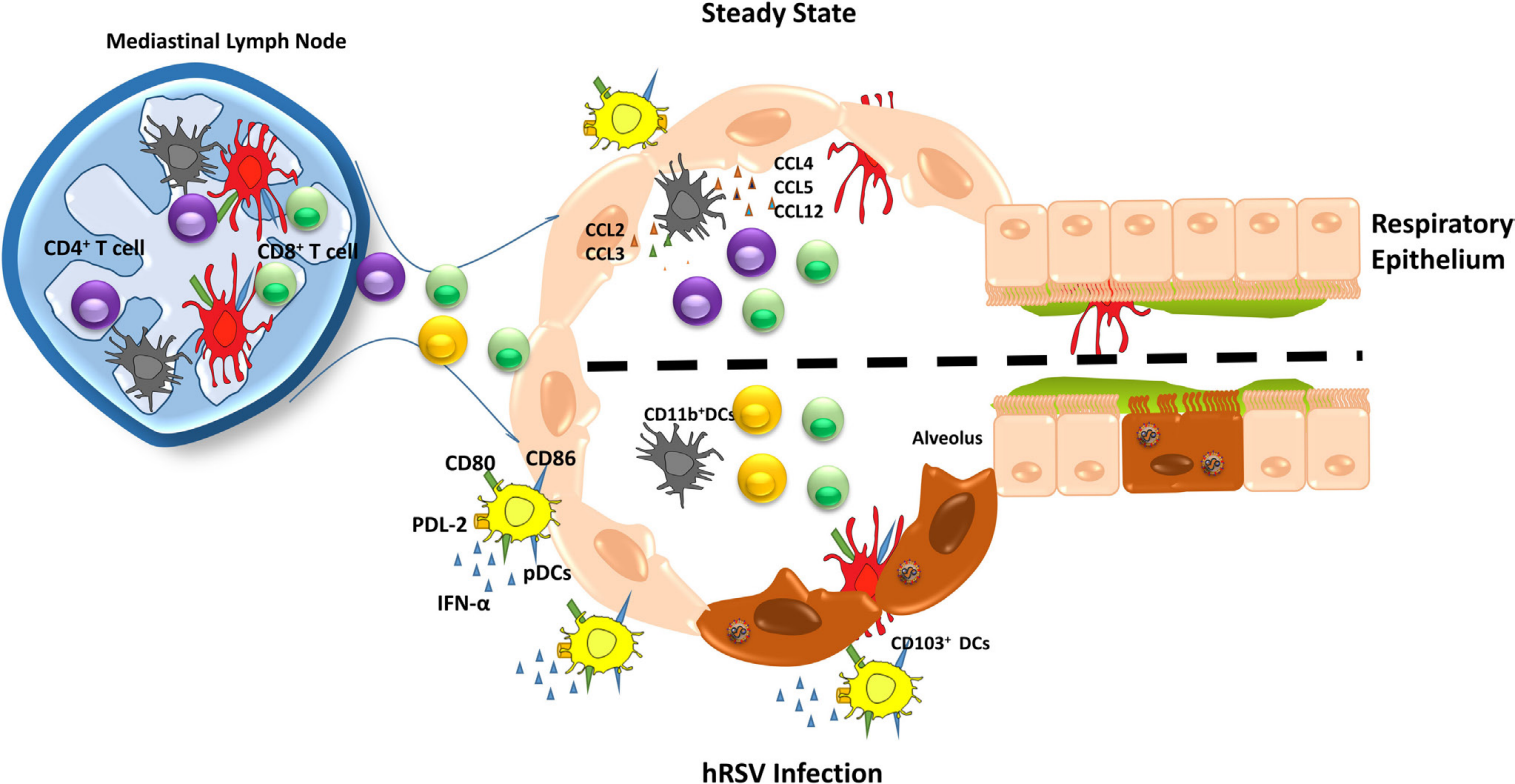
Different roles of dendritic cells (DCs) subsets during human respiratory syncytial virus (hRSV) infection. During the steady state (upper), DCs subset maintain the lung homeostasis and when an infection occurs, they are capable to migrate to mediastinal lymph node to present antigens to T cells, promoting an immune response. Upon hRSV infection, plasmacytoid DCs (pDCs) increase in numbers, as well as the expression of co-stimulatory molecules, such as CD80, CD86. In addition, pDCs secretes high levels of IFN-α and PDL-2 expression is reduced. Moreover, in response to hRSV infection, CD11b^+^ DCs fail to activate CD4^+^ T cells leading a poor response against the virus. On the other hand, CD103^+^ DCs show a lower expression of CD80 and CD86, which are required for an appropriate antiviral immune response.

CD103^+^ cDCs are the most prevalent population of DCs in the lungs, which locate directly underneath the airway epithelium (14). CD103^+^ cDCs express the integrin α_E_β_7_ and are found mainly at the lamina basal of the bronchial epithelia and arterioles (106). This DCs subset efficiently loads virus-derived peptides onto MHC-I molecules, inducing a potent proliferation of naïve CD8^+^ T cells (Figure 3) (107). Therefore, CD103^+^ cDCs work as key mediators of immunity to intracellular pathogens infecting the lungs (108, 109). In several studies with hRSV, neonatal mice have been used to better compare the human clinical features with mouse models of the disease (110). CD103^+^ DCs from neonatal mice infected with hRSV showed lower expression of co-stimulatory molecules, CD80 and CD86, as compared to the adult counterparts, affecting the T cell synapsis quality (110). For this reason, neonatal mice infected with hRSV generated a distinct CD8^+^ T cell response as compared to adult mice, suggesting a key role of CD103^+^ DCs (110). Furthermore, the immunization with F virus-like particles (VLP) of hRSV-infected mice showed high levels of CD103^+^ DCs in bronchoalveolar lavage fluids (BALFs) and lungs (23). Moreover, the mediastinal LN from F VLP-immunized mice showed higher levels of CD103^+^ DCs and resident CD8α^+^ DCs (23). It is known that after hRSV infection is resolved, is possible to develop subsequent asthma during childhood (111, 112). Here, CD103^+^ DCs play a protective role during asthma/allergic-related symptoms by producing IL-12 (113).

As mentioned above, another DCs cell subset, CD11b^+^ cDCs locate in the lung parenchyma (114, 115). In addition to the uptake of extracellular pathogens, the main function of these cells is to present antigens to CD4^+^ T cells (116). Studies were carried out in mice to understand the role of CD11b^+^ DCs during hRSV infection. In lungs from mice infected with hRSV, an increase was observed for the frequency of CD11b^+^ DCs (114). Moreover, it was determined that the ability of CD11b^+^ DCs to migrate to LN remained intact (114). One of the mechanisms proposed for the accumulation of CD11b^+^ DCs in the airways is that during hRSV infection, the high levels detected for CCL20 in the lungs can attract CD11b^+^ CCR6^+^ DCs (117, 118). To evaluate the role of CD11b^+^ CCR6^+^ DCs during hRSV infection, studies were performed in CCR6-deficient mice, showing an increase of viral clearance, lower levels of Th2 pro-inflammatory cytokines, as well as reduced mucus production (117, 118). Overall, these findings suggest that the CD11b^+^ CCR6^+^ DCs promote a Th2 immune response upon hRSV infection (117, 118). Additional studies performed in the neonatal murine model described that lung CD11b^+^ DCs express higher levels of the IL-4 α receptor (IL-4Rα) as compared to adult mice (119). *In vitro* experiments using neonatal murine CD11b^+^ DCs showed that IL-4Rα promotes the differentiation of T cells into a Th2 phenotype (119). Furthermore, when the IL-4Rα was deleted, maturation of these cells increased, suggesting that neonatal CD11b^+^ DCs are less prone to maturation. This feature of neonatal DCs could impair their capacity to induce a protective hRSV-specific immune response (119). Similar results were observed *in vivo* experiments; however, the deletion of IL-4Rα could be observed in several cells types including AMs, CD11b^+^ DCs, and CD103^+^ DCs (119).

Plasmacytoid DCs are the other prominent subset present in the lungs, which are an important source of IFN-α/β, fundamental antiviral cytokines during an infection, both in humans and mice (120, 121). The role of this DCs subset was elucidated by performing experiments with hRSV-infected bone marrow-derived pDCs, which expressed high levels of IFN-α, CD80, and CD86 and lower PD-L2 levels (Figure 3) (122). Moreover, in the same study, authors showed that the number of pDCs was increased early after infection and then decreased with the resolution of the disease, suggesting that pDCs are required during early stages of infection by hRSV (122). Also, it has been demonstrated that when pDCs are depleted using a 120G8 antibody, the lungs of hRSV-infected mice displayed an enhanced inflammation consisting mainly of mononuclear cells and lymphocytes, together an increase of viral loads (123). Consistently with these findings, it was observed that BALFs from hRSV-infected preterm born children showed reduced recruitment of pDCs into the lungs. These data are in agreement with the notion that low numbers of pDCs could work as a risk factor for severe bronchiolitis (124).

Additionally, three pDCs subsets have been characterized that include CD8α^−^β^−^, CD8α^+^β^−^, and CD8α^+^β^+^ (125). The frequency of these subsets is 61, 22, and 6% in the lungs of healthy mice, respectively (125). However, beside all the current knowledge about the role of the different subset of DCs during hRSV infection, there are no data about the contribution of these cells to the hRSV-induced pathology.

In summary, all DCs subsets seem to be important for an antiviral immune response. However, hRSV is able to modulate the function of these cells by promoting an imbalance between these subsets, which could be critical for the resolution of the disease caused by this virus.

## Concluding Remarks

The MPS consists of a family of cells that include monocytes, MΦ, and DCs, among others (1–3). These cells are characterized by their high mobility, phagocytic capacity, and ability to secrete a broad spectrum of immunomodulatory molecules (2). MPS exerts several functions in health and disease in several tissues (2). In the lungs, MPS plays an important role in the maintenance of homeostasis during steady state. During an infection, the MPS works as the first line of immune response against pathogens (2). Among the respiratory pathogens, hRSV is considered the most important cause of respiratory illness in infants and young children (33). The main severe clinical manifestation due to the hRSV infection is bronchiolitis (33). In this context, the MPS can contribute to the development of the immunopathology induced by hRSV (10). This viral pathogen is able to infect the MPS cells, altering the proper immune response required for viral clearance and the later acquisition of an antiviral immune memory (10). During the acute phase of the hRSV infection, monocytes are important to initiate the innate immune response, secreting cytokines and chemokines that recruit other immune cells, such as MΦ, eosinophils, and neutrophils (126). The normal function of monocytes during hRSV infection can be impaired not only by the pathogen itself but also by the contact with virus-infected AECs, contributing to the development of severe bronchiolitis (62).

During hRSV infection, MΦs play a protective role against infection and, finally, these cells are required for proper virus clearance (77). This notion is supported by experimental data obtained from various experimental models, such as neonatal mice and MΦ-depleted animals.

One of the effector mechanisms of MΦs is the secretion of IFN-γ and IL-12, which are reduced in human neonates infected by hRSV, contributing to development of bronchiolitis (81). Also, the inefficient recruitment of T and NKT cells into the airways (91) contributes to the development of bronchiolitis. As a consequence, there is a significant decrease of the protective capacity of the immune response triggered by hRSV and promoting virus spreading (91).

The ability of hRSV to infect DCs seems to be a major virulence mechanism used by this pathogen. The hRSV-infection of DCs impairs the correct immunological synapsis, which is required for T cell activation (104). These findings may contribute to explaining the lack of an effective immunological memory against hRSV, which allow subsequent reinfections throughout life. Moreover, pDCs are the major source of type I IFNs, cytokines necessary to induce an appropriate antiviral immune response (122). Consistently with a lack of protective immunity to hRSV, DCs infected by this virus show an impaired capacity to produce type I IFNs (122). Based on the current knowledge relative to the role of DCs in the hRSV-infection, these cells could be important for the promotion of an exacerbation of the inflammation during bronchiolitis. Besides the actual knowledge about the MPS in the hRSV immunopathology, is still necessary to further understand the mechanisms involved in the impairment of MPS function by hRSV virulence factors.

## Author Contributions

AK wrote, revised, and edited the article and figures. KB, JE, RP, and EJ wrote the article and drew the figures.

## Conflict of Interest Statement

The authors declare that the research was conducted in the absence of any commercial or financial relationships that could be construed as a potential conflict of interest.
